# Confluent and Reticulated Papillomatosis Associated with Polycystic Ovarian Syndrome

**DOI:** 10.7759/cureus.3956

**Published:** 2019-01-25

**Authors:** Pallavi Basu, Philip R Cohen

**Affiliations:** 1 Dermatology, University of California, San Diego, USA; 2 Dermatology, San Diego Family Dermatology, San Diego, USA

**Keywords:** acanthosis, confluent, insulin, nigricans, ovary, papillomatosis, polycystic, resistance, reticulated, syndrome

## Abstract

Polycystic ovarian syndrome is an endocrine disorder diagnosed commonly in young women. Various cutaneous manifestations can include acanthosis nigricans, acne, hirsutism, and alopecia. Confluent and reticulated papillomatosis is a rare skin condition that may be associated with polycystic ovarian syndrome. The etiology of confluent and reticulated papillomatosis is not yet well established but multiple theories exist regarding its pathogenesis. We describe a woman with established polycystic ovarian syndrome who presented with confluent and reticulated papillomatosis; her skin condition was successfully treated with azithromycin. The clinical features, differential diagnosis, epidemiology, and proposed etiologies for confluent and reticulated papillomatosis are discussed as well as possible treatment options. Among women with polycystic ovarian syndrome, confluent and reticulated papillomatosis and acanthosis nigricans can occur concurrently. Additionally, it is possible that confluent and reticulated papillomatosis occurs more commonly in this patient population.

## Introduction

Polycystic ovarian syndrome (PCOS) is a common endocrine disorder affecting women of reproductive age. It is defined by the presence of two out of three of the Rotterdam criteria: oligo- or anovulation, clinical and/or biochemical signs of hyperandrogenism, and polycystic ovaries on ultrasound. Exclusion of other etiologies, such as congenital adrenal hyperplasia, androgen-secreting tumors, and Cushing’s syndrome, is also required [1, 2]. The cutaneous manifestations of PCOS may include acanthosis nigricans and possible sequelae of hyperandrogenism – acne, androgenic alopecia, hirsutism, and seborrhea. Less commonly, confluent and reticulated papillomatosis (CARP) can be observed in patients with PCOS or history of insulin resistance [3-6]. We present a woman who has CARP associated with PCOS and discuss the utility of azithromycin in treating this skin condition.

## Case presentation

A 31-year-old Hispanic woman presented for an evaluation of a diffuse asymptomatic rash, of four years duration, on her anterior neck and her chest between her breasts. Her medical history was significant for PCOS diagnosed 10 years earlier. Clinical manifestations of her PCOS included heavy, irregular menses, acne, hirsutism, and acanthosis nigricans. Her medication only included an oral contraceptive pill.

**Figure 1:**
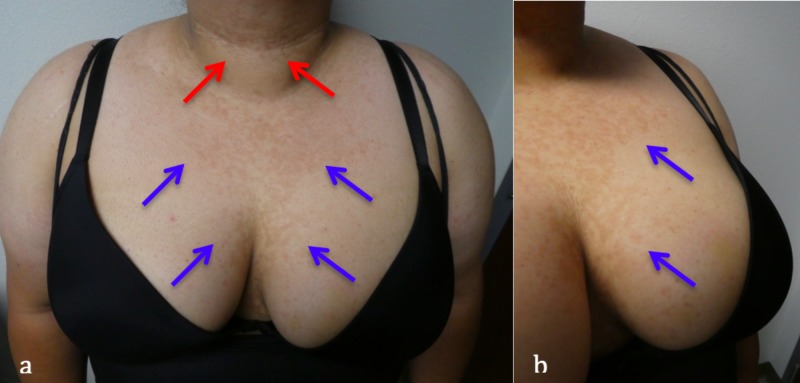
Confluent and reticulated papillomatosis in a woman with polycystic ovarian syndrome. Distant (a) and close-up (b) views of a 31-year-old Hispanic woman with hyperpigmented and reticulated macules and patches on her central chest (blue arrows) and anterior neck (red arrows).

**Figure 2:**
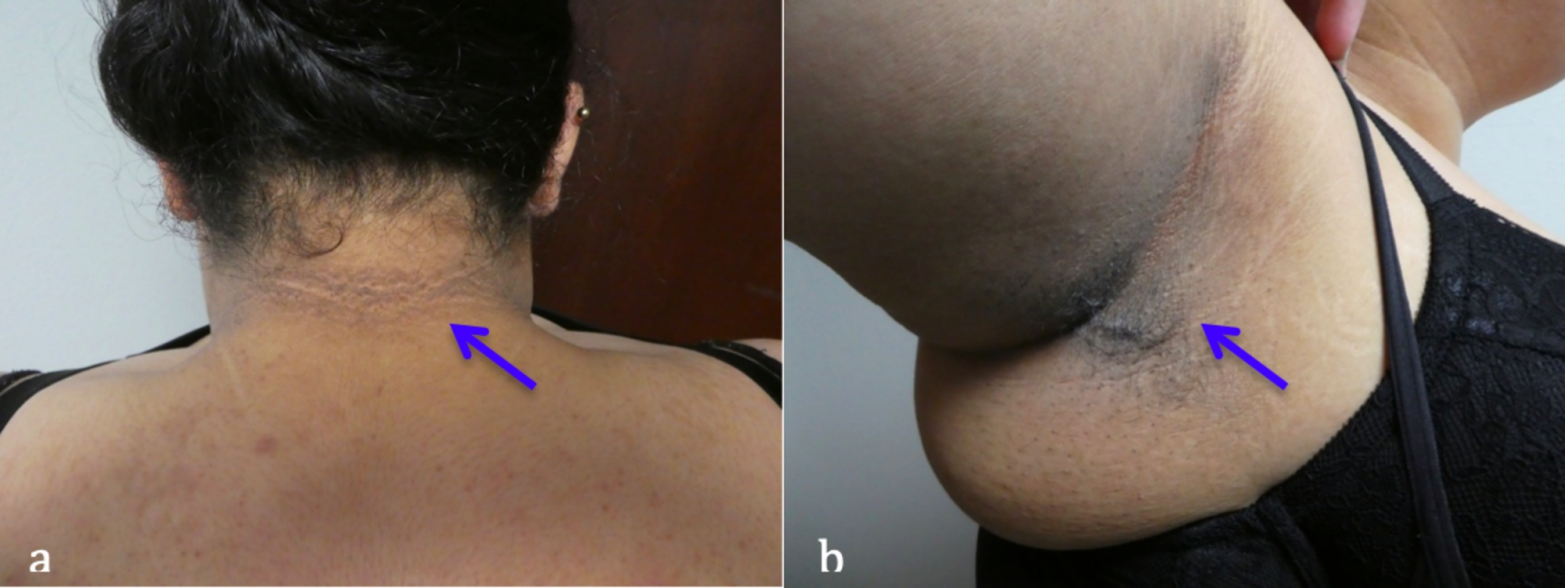
Acanthosis nigricans concurrently present in a woman with confluent and reticulated papillomatosis and polycystic ovarian syndrome. Hyperpigmented plaques (blue arrows) consistent with acanthosis nigricans located on the (a) posterior neck and (b) left axilla of a 31-year-old Hispanic woman.

Cutaneous examination, on initial presentation, revealed reticulated hyperpigmented patches on her chest – in between her breasts – and anterior neck (Figure 1). In addition, there was not only macular hyperpigmentation on her cheeks (consistent with melasma), but also velvet-like hyperpigmented plaques on her posterior neck and axilla (consistent with acanthosis nigricans) (Figure 2).

Correlation of the presentation and clinical examination of the anterior neck and chest lesions was most consistent with CARP. Azithromycin, 250 mg once a day, was prescribed.

**Figure 3:**
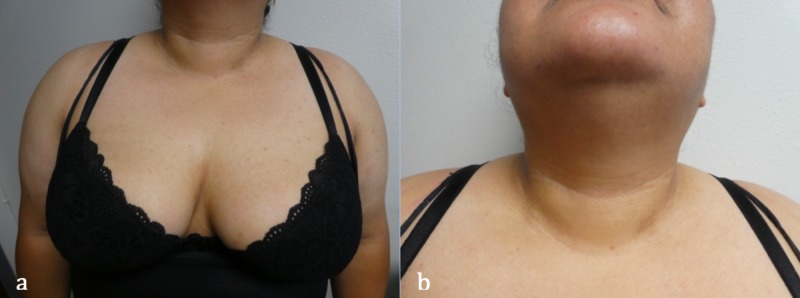
Successful treatment of confluent and reticulated papillomatosis with daily azithromycin. Central chest (a) and anterior neck (b) of a 31-year-old Hispanic woman show complete resolution of the reticulated hyperpigmentation following two months of daily treatment with 250 mg of azithromycin.

After two months of treatment, her chest and anterior neck dermatosis had resolved; the plaques had flattened and the skin hyperpigmentation had faded (Figure 3). The velvet-like plaques on her posterior neck and axillae persisted. The clinical response to azithromycin confirmed the suspected diagnosis of CARP on her chest and anterior neck. In contrast, the persistence of the lesions on her posterior neck and axillae strongly suggested a diagnosis of acanthosis nigricans at these sites.

The dose of azithromycin was titrated down and eventually stopped during the next month. There was no recurrence of the CARP on subsequent follow-up visits.

## Discussion

Confluent and reticulated papillomatosis is characterized by hyperkeratotic, verrucous papules which have a reticular quality peripherally and coalesce into plaques centrally [7]. The number of papules typically can progress over months to years and range in color from pink to brown. However, hypopigmented lesions have also been observed in dark-skinned individuals [8].

Lesions are located most commonly on the trunk – specifically the chest, abdomen, and back – though other areas have been reported [6]. The differential diagnosis of confluent and reticulated papillomatosis may include acanthosis nigricans, Darier disease, Dowling-Degos disease, dyskeratosis congenita, Galli-Galli disease, macular amyloidosis, and tinea versicolor. Distinguishing features among these differential diagnoses are presented in Table 1.

**Table 1:** Clinical differential diagnosis of confluent and reticulated papillomatosis.

Diagnosis	Clinical characteristics
Acanthosis nigricans	Velvet-like, hyperpigmented plaques have a predilection for flexural areas.
Confluent and reticulated papillomatosis	Hyperpigmented scaly macules or papillomatous papules coalesce into confluent patches or plaques centrally and exhibit a reticular pattern peripherally. Rarely, they can appear as atrophic macules with a cigarette paper-like surface.
Darier disease	Inheritance is autosomal dominant with nail, skin, and mucous membrane findings. Yellow-brown, skin-colored or hyperpigmented waxy papules may appear on the chest and back that coalesce into crusted plaques. Groin, axilla, and inframammary involvement is often present.
Dowling-Degos disease	Inheritance is autosomal dominant with reticular hyperpigmentation beginning in the axillae and groin. Other body folds can later become involved (intergluteal and inframammary folds, neck, and inner aspects of the arms and thighs).
Dyskeratosis congenita	A triad of mucocutaneous findings is present: abnormal skin pigmentation (lacy reticular hyperpigmentation of the upper chest and back), nail dystrophy (thin nail plates with longitudinal ridges disappearing with age), and oral leukoplakia.
Galli-Galli disease	Inheritance is autosomal dominant and appears similar to Dowling-Degos but with unique histological differences (suprabasal nondyskeratotic acantholysis).
Macular amyloidosis	Pruritic, brown, rippled macules on the mid-upper back can present with or without findings of systemic amyloidosis. Skin biopsy with amyloid staining is positive.
Tinea versicolor	Hyperpigmented macules or patches appear on the trunk or proximal upper extremities. A potassium hydroxide preparation of the scale will reveal hyphae and yeast cells.

CARP is most prevalent amongst teenagers and is not known to have any particular geographic distribution. A sex preference has not been established. However, one case series suggested a higher prevalence in men [9, 10].

Histologic features of CARP include hyperkeratosis, papillomatosis, and acanthosis with mildly to moderately increased pigmentation [3, 9]. Dermal ectasia and perivascular lymphocytic inflammation have also been noted. *Pityrosporum* yeast forms may or may not be present. The presence of yeast may explain the successful treatment of CARP with antifungals. However, in some individuals, it is unclear whether this is due to treatment of overlying tinea versicolor [9].

Multiple hypotheses exist regarding the pathogenesis of CARP. Most attribute the disorder to be related to keratinization given the histologic features and clinical response to retinoids and vitamin D-derived therapies [6]. However, the response to antibiotic therapy suggests that bacterial pathogens may be involved in the development of CARP. One hypothesis proposes direct inoculation with a single pathogen while another suggests an alteration of sebum by host bacteria or abnormal response to host bacteria, given CARP’s preference for seborrheic areas and occurrence in adolescents [11, 12].

Metabolic abnormalities – particularly impaired glucose tolerance, hyperinsulinemia, hypothyroidism and diabetes mellitus – have been observed in patients with CARP. Other studies have also reported the presence of both acanthosis nigricans and CARP concurrently in patients with metabolic dysfunction [3-5, 13]. Women with CARP and concurrent PCOS or insulin resistance, including our patient, are summarized in Table 2 [3, 4, 6].

**Table 2:** Confluent and reticulated papillomatosis in patients with polycystic ovarian syndrome or insulin resistance. AN: Acanthosis nigricans; BID: Twice daily; C: Case; CARP: Confluent and reticulated papillomatosis; CR: Current report; dx: Diagnosis; IR: Insulin resistance; mg: Milligrams; PCOS: Polycystic ovarian syndrome; tx: Treatment; y: Years; +: Present; -: Absent

C	CARP dx age (y)	PCOS/IR dx age (y)	AN (+/-)	CARP location	Treatment	Response to tx	Ref
1	13	19, PCOS	+	Abdomen, back, chest	Azithromycin 500 mg three times weekly	Complete resolution	[6]
2	14	21, PCOS	-	Chest, face	Drospirenone	Complete resolution	[4]
3	19	19, IR	+	Abdomen, back, chest	Oral minocycline 100 mg BID with topical calcipotriol for two months	No change after two months; however, four months later, skin improved	[3]
4	31	21, PCOS	+	Anterior neck, chest	Azithromycin 250 mg once a day	Complete resolution	CR

The age of CARP diagnosis in women with PCOS or insulin resistance ranged from 13 years to 31 years (median 16.5 years). Among this patient population, the age range of PCOS diagnosis ranged from 19 to 21 years of age (median 20 years). The diagnosis of CARP preceded the diagnosis of PCOS in two of the women by either six or seven years. Diagnoses of CARP and insulin resistance occurred simultaneously in one woman, and CARP was diagnosed 10 years after the diagnosis of PCOS in the fourth woman.

CARP lesions localized to the chest in all four women with PCOS or insulin resistance. The abdomen and back were also simultaneously involved in two out of four (50%) of the women. The anterior neck was involved exclusively in our patient, and facial lesions of CARP were present in one woman. Three out of four (75%) of the women presented with acanthosis nigricans concurrently.

Two out of four (50%) of the women were treated with azithromycin. Both experienced complete resolution following two months of treatment. Another woman was treated with drospirenone and her lesions eventually completely cleared. One woman was started on a combination of oral minocycline and topical calcitriol; initial evaluation, after two months of treatment, did not show any change in her CARP lesions. However, significant improvement of her skin was observed – in association with a weight loss of ten kilograms – at her six month follow-up visit.

In addition to the four women with PCOS or insulin resistance associated with CARP, CARP has also been described in a 17-year-old man in whom insulin resistance was diagnosed two years later [5]. His CARP lesions were located on the chest and abdomen, back (upper, lumbar and sacral regions), and upper arms. He also had concurrent acanthosis nigricans. He was treated with etretinate, 40 mg daily; however, he developed liver dysfunction (that was attributed to the medication) and therapy was discontinued.

It is possible that CARP may be more common in individuals with PCOS or insulin resistance than is established in the literature. Supporting evidence for this claim includes that both acanthosis nigricans and CARP are disorders of keratinization, though histologically distinct. Additionally, some investigators suggest common predisposing factors in acanthosis nigricans and CARP among patients with obesity [5].

Indeed, the etiology of acanthosis and CARP may overlap. Researchers have proposed that the high levels of insulin result in inappropriate activation of cellular receptors promoting epidermal proliferation and papillomatosis [3, 14]. This mechanism may represent a common pathway that acts in the pathogenesis of both acanthosis nigricans and CARP [3].

Medications such as metformin may be useful in the management of CARP depending on the extent that the condition is associated with insulin resistance. However, CARP in our patient – who was not on metformin – responded to antibiotic treatment. Her successful management with azithromycin emphasizes the possibility that bacterial inoculation – alone or as an etiologic cofactor – plays a role in CARP pathogenesis.

A variety of treatment options exist for the management of CARP, including antibiotics, retinoids, and other miscellaneous therapies [6]. Possible antibiotics include azithromycin, clarithromycin, doxycycline, erythromycin, fusidic aid, minocycline, and roxithromycin. Retinoids that have been successfully used include either oral agents such as isotretinoin or topical agents such as tazarotene and tretinoin. Other therapies that have been efficacious include calcipotriol, ketoconazole cream, oral contraceptives, selenium sulfide lotion, and tacalcitol; however, the mechanisms of action underlying improvement are not well understood.

Antibiotics may be beneficial in treating CARP not for their antimicrobial properties but for their anti-inflammatory and immunosuppressive effects. Tetracyclines at lower doses are effective in treating inflammatory skin conditions [15]. Macrolide antibiotics have been shown to modulate the production of inflammatory cytokines [16]. However, it is also possible that these anti-inflammatory properties may not have a particularly salient role in CARP as inflammation is minimal based upon the microscopic features of lesion biopsy specimens [17].

Minocycline is the most common antibiotic used for treating CARP. However, the efficacy of azithromycin has also been reported for CARP treatment [6]. Our patient was treated with azithromycin since minocycline has more potential drug-associated adverse side effects. She experienced over 95% clearing of her CARP lesions by her one-month follow-up visit and complete resolution of her skin condition after two months of therapy.

## Conclusions

CARP is a rare condition whose etiology has not been definitely established. Pathogenesis may be multifactorial since it occurs not only in healthy individuals but also in patients with obesity, insulin resistance, and/or PCOS. There are several agents that have been successfully used for the management of CARP; however, minocycline or azithromycin are usually considered to be first-line therapies. In women with PCOS, CARP may not only occur more commonly, but also present with acanthosis nigricans simultaneously.
